# Light Chain Deposition Disease: A Morphological Case Report

**DOI:** 10.7759/cureus.26357

**Published:** 2022-06-27

**Authors:** Hristo Popov, George S Stoyanov, Peter Ghenev

**Affiliations:** 1 General and Clinical Pathology/Forensic Medicine and Deontology, Medical University of Varna, Varna, BGR; 2 General and Clinical Pathology, St. Marina University Hospital, Varna, BGR

**Keywords:** multiple myeloma, case report, kidney, nephropathology, monoclonal immunoglobulin deposition disease, light chain deposition disease

## Abstract

Light chain deposition disease (LCDD) is a rare condition associated with the overproduction and deposition of monoclonal light chain immunoglobulins. The kidneys are universally affected in LCDD, with the development of renal failure with nephrotic syndrome, microscopic hematuria, and proteinuria. Most cases are associated with a plasma cell neoplasm (multiple myeloma or plasmacytoma) or other lymphoproliferative disorders, with a reasonable number of cases also being idiopathic. Other organs can be affected in rare cases, without producing significant symptoms, predominantly the liver and heart. In this report, we discuss a case of a 72-year-old female presenting with the aforementioned symptoms. Percutaneous ultrasound-guided kidney biopsy revealed nodular sclerosis with periodic acid-Schiff stain (PAS)-positive, Congo red-negative, silver impregnation weakly-positive, and Masson's trichrome-positive (red reaction) deposits with ribbon-like changes together with light chain deposits in the tubular basement membranes. As LCDD was diagnosed, the patient was subjected to further tests, with multiple myeloma of the thoracic vertebrae also being diagnosed. The patient was started on myeloma treatment; however, she subsequently developed a severe lower limb infection that required amputation, after which she developed sepsis and expired.

## Introduction

Light chain deposition disease (LCDD) is the most common variant of monoclonal immunoglobulin deposition disease, which is characterized by light chain immunoglobulin deposition in the tissues of the body [1]. While the immunoglobulin deposition may be diffuse, the kidneys are always affected, with only a minority of cases developing extrarenal involvement [2].

The condition was first described in 1976 in two patients with end-stage renal disease and, due to its rarity, its exact incidence is unknown in the general population [3]. Based on the largest collected cohort to date by Pozzi et al., involving an analysis of 63 patients, the average age of diagnosis is 58 years, with other smaller studies estimating it as follows: 56 years, Sayed et at. in 53 patients; 51 years, Li et al. in 48 patients; and 56 years, Nasr et al. in 64 patients (some of which are with heavy chain deposition), with males being affected more often than females [2,4-6].

LCDD develops due to the overproduction and thereby the deposition of abnormal immunoglobulins, with around 60% of cases developing in the context of multiple myeloma/plasmacytoma and other lymphoproliferative disorders; however, in a large number of cases (up to 30% in some studies), an underlying cause cannot be identified - idiopathic LCDD [2]. Light chain deposits can develop in any other extrarenal site but are usually rare and characterized by an indolent clinical course, the liver and heart being the most common secondary sites [7]. Furthermore, some patients have concomitant cast nephropathy caused by the monoclonal proteins, leading to acute renal damage. Clinically, LCDD presents with renal failure, proteinuria, microscopic hematuria, and nephrotic syndrome [8].

## Case presentation

We report the histopathological findings in a 72-year-old female presenting with nephrotic syndrome, long-term hypertension, pretibial edema, and grade II obesity. Urinalysis reported proteinuria (5.5 grams per liter), microscopic hematuria, and creatinine of 940 µmol per liter. Blood tests reported hemoglobin levels of 96 g/l (anemia), and all other results were within the reference range. Based on her symptoms and laboratory findings, the patient was scheduled for a percutaneous kidney biopsy with a 16-G needle under ultrasound guidance.

**Figure 1:**
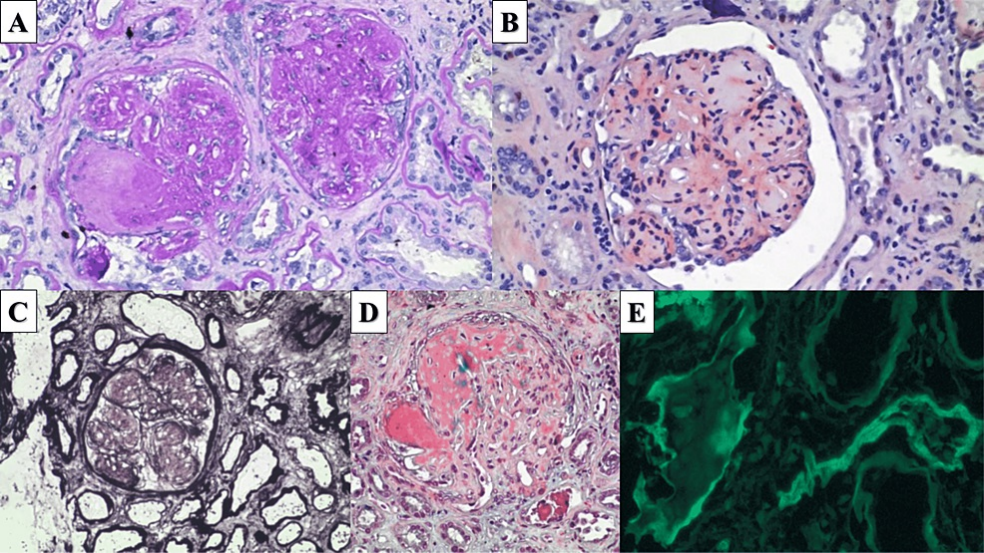
Late LCDD presenting as nodular glomerulosclerosis A: PAS-positive nodular deposits (PAS stain, original magnification 400x). B: The same nodules are Congo-negative (Congo red stain, original magnification 400x). C: The same nodules are weakly positive for silver impregnation, a strong reaction is noted in the basal membrane of the tubular epithelium with ribbon-like change [Silver impregnation (modified method), original magnification 400x]. D: Red reaction in nodular glomerular deposits (Masson's trichrome, original magnification 400x). E: Lineal light chain deposition in the tubular basal membranes (direct immunofluorescence, original magnification 400x) LCDD: light chain deposition disease; PAS: periodic acid-Schiff stain

Histology and histochemistry initially showed nodular sclerosis with mesangial deposits being periodic acid-Schiff stain (PAS)-positive, Congo red-negative, and having a weak reaction to silver impregnation, which also underlined ribbon-like thickened tubular basement membranes (Figures 1A-1C). The mesangial deposits were also Masson's trichrome-positive (red reaction), suggesting their immunoglobulin nature (Figure 1D). Immunofluorescence showed that the deposits in the tubular basement membranes were comprised of lambda chains (Figure 1E).

Based on the morphological findings, the diagnosis of LCDD was established. Secondary urinary analysis revealed Bence-Jones protein in the urine and the patient was scheduled for whole-body CT to establish any foci suggestive of lymphoproliferative disorder. The CT showed osteolytic foci in the fifth, sixth, and seventh thoracic vertebrae. Secondary biopsy of the osteolytic lesions showed a diffuse plasma cell proliferation, positive for cluster of differentiation (CD) 138, and the diagnosis of multiple myeloma with secondary LCDD was established. The patient was started on treatment with bortezomib, dexamethasone, and darbepoetin alfa. However, she developed deep vein thrombosis with an associated severe subcutaneous infection in her left lower limb, requiring distal amputation. Microbiology revealed *Staphylococcus epidermidis*. However, despite sufficient amputation and antimicrobial treatment with cefazolin and metronidazole, the patient expired during the early postoperative period due to developing sepsis three months after the initial diagnosis of LCDD.

## Discussion

Under light microscopy, early changes in LCDD show discrete mesangial enlargement, with a negative reaction for Congo red, while advanced changes present with nodular sclerosis very similar to type III diabetic nephropathy - Kimmelstiel-Wilson type. Immunofluorescence testing with kappa and lambda chains shows deposits in the mesangium of the glomeruli and along the tubular basement membranes [9]. Under electron microscopy, powdery deposits similar to ground pepper are observed in the basal lamina of the glomerular capillaries and the tubular basal membrane [9].

Due to the condition's rarity and morphological similarities with other glomerular lesions, the differential diagnosis is challenging and involves a broad set of conditions: diabetic nephropathy, idiopathic nodular glomerulosclerosis, amyloidosis, and cryoglobulinemia, with differential histochemical features as shown in Table 1 [9].

**Table 1:** Differential diagnosis and special stain reactions in nodular glomerulosclerosis LCDD: light chain deposition disease; PAS: periodic acid-Schiff stain

Condition	PAS reaction	Silver impregnation	Congo red	Masson's trichrome	Immunofluorescence
Nodular diabetic glomerulosclerosis	Positive	Positive	Negative	Green reaction	Linear IgG or negative
Idiopathic nodular glomerulosclerosis	Positive	Positive	Negative	Green reaction	Negative
LCDD	Positive	Negative to weak positive	Negative	Red reaction	Kappa/lambda
Amyloidosis	Weak to moderately positive	Negative	Positive	Negative	Positive
Cryoglobulinemia	Positive	Positive subendothelial deposits	Negative	Red reaction	IgG, IgM, C1q, C3, kappa, and lambda

Immunofluorescence is key in differentiating these conditions, with histochemical reactions being suggestive but not definitive [2,3,7,9].

Clinically, LCDD usually presents with hypertension, microhematuria, and proteinuria in the absence of therapy and its clinical course is one of rapidly progressing chronic kidney disease requiring renal replacement therapy [10]. In the idiopathic form, if a kidney transplant is performed in such cases, the outcomes are generally poor and associated with allograft LCDD, which occurs within a few years of transplantation [11,12].

Based on its broad differential diagnosis and its usual age of onset, LCDD can often be misdiagnosed and therefore underreported. This likelihood is especially high when the patients have concomitant diabetes or new-onset kidney failure in the context of known lymphoproliferative disorders, when it can be interpreted as disease progression or secondary renal involvement by conventional mechanisms.

## Conclusions

LCDD is a rare condition affecting the kidney and, in rare cases, other organs; it is characterized by lambda light chains being deposited in the glomeruli and basal membrane of the tubular system. Although most commonly associated with multiple myeloma and lymphomas, some cases can be idiopathic. As seen in our case, the renal biopsy, if interpreted properly, can guide toward a detailed exploration and identification of the primary disease, which turned out to be multiple myeloma in our case.
